# Nonlinear analysis of neuronal firing modulated by sinusoidal stimulation at axons in rat hippocampus

**DOI:** 10.3389/fncom.2024.1388224

**Published:** 2024-08-30

**Authors:** Yue Yuan, Xiangyu Ye, Jian Cui, Junyang Zhang, Zhaoxiang Wang

**Affiliations:** ^1^Zhejiang Lab, Hangzhou, China; ^2^Key Laboratory for Biomedical Engineering of Ministry of Education, College of Biomedical Engineering and Instrument Science, Zhejiang University, Hangzhou, China

**Keywords:** sinusoidal stimulation, unit spike, fractal, long-range correlations, Fano factor, Hurst exponents, hippocampus

## Abstract

**Introduction:**

Electrical stimulation of the brain has shown promising prospects in treating various brain diseases. Although biphasic pulse stimulation remains the predominant clinical approach, there has been increasing interest in exploring alternative stimulation waveforms, such as sinusoidal stimulation, to improve the effectiveness of brain stimulation and to expand its application to a wider range of brain disorders. Despite this growing attention, the effects of sinusoidal stimulation on neurons, especially on their nonlinear firing characteristics, remains unclear.

**Methods:**

To address the question, 50 Hz sinusoidal stimulation was applied on Schaffer collaterals of the rat hippocampal CA1 region *in vivo*. Single unit activity of both pyramidal cells and interneurons in the downstream CA1 region was recorded and analyzed. Two fractal indexes, namely the Fano factor and Hurst exponent, were used to evaluate changes in the long-range correlations, a manifestation of nonlinear dynamics, in spike sequences of neuronal firing.

**Results:**

The results demonstrate that sinusoidal electrical stimulation increased the firing rates of both pyramidal cells and interneurons, as well as altered their firing to stimulation-related patterns. Importantly, the sinusoidal stimulation increased, rather than decreased the scaling exponents of both Fano factor and Hurst exponent, indicating an increase in the long-range correlations of both pyramidal cells and interneurons.

**Discussion:**

The results firstly reported that periodic sinusoidal stimulation without long-range correlations can increase the long-range correlations of neurons in the downstream post-synaptic area. These results provide new nonlinear mechanisms of brain sinusoidal stimulation and facilitate the development of new stimulation modes.

## 1 Introduction

Electrical stimulation (ES) has emerged as an increasingly attractive therapeutic option for various neurological and psychiatric disorders (Lozano et al., 2019; Krauss et al., 2021). Typically, clinical ES usually utilizes charge-balanced square pulse waveforms. However, alternative electrical waveforms such as sinusoids have garnered attention due to their potential benefits in terms of therapeutic efficacy and safety (Foutz and McIntyre, 2010; Mottaghi et al., 2020; Krauss et al., 2021). Sinusoidal stimulation has been utilized in retinal stimulation for the restoration of visual function (Twyford and Fried, 2016; Su et al., 2022), as well as in in transcranial electrical stimulation (TES) for conditions such as Parkinson’s disease, tremor, schizophrenia, and obsessive-compulsive disorder (Antal and Paulus, 2013; Brittain et al., 2013; Liu et al., 2021). It has also shown promise in deep brain stimulation (DBS) for Parkinson’s disease and epilepsy (Lian et al., 2003; Guo et al., 2016; Liu et al., 2018; Mottaghi et al., 2020), as well as in spinal cord stimulation (SCS) for pain management (Soin et al., 2015). Furthermore, sinusoidal stimulation has also been reported to induce conduction block in fibers, which is believed to underlie the therapeutic effects of many electrical stimulations (Wodlinger et al., 2013; Fisher and Velasco, 2014). Previous studies have also demonstrated that sinusoidal waveforms may activate neurons at a considerably lower electrical intensity compared to pulse stimulation, thereby reducing the risk of tissue damage and extending the lifespan of stimulation batteries (Francis et al., 2003; Foutz and McIntyre, 2010; Lin et al., 2013). However, the effects of sinusoidal stimulation on neuronal activity remain unclear.

The neuronal firing activity exhibits distinct nonlinear dynamic properties, including fractal, bifurcation, and chaos (Teich, 1989; Fan and Holden, 1993; Munn et al., 2020). Fractal theory, initially devised to describe the complex geometry of coastlines, was later extended by Mandelbrot to analyze time series data. Since then, fractal analysis has been widely utilized in neurophysiological research (Mandelbrot, 1982; Teich et al., 1997; Lowen et al., 1999; Das et al., 2003; Chen et al., 2015; Munn et al., 2020). In the time domain, fractal is characterized by long-range correlations among events that spanning multiple time scales (Teich et al., 1997; Munn et al., 2020). For example, a fractal firing sequences of neuronal spikes exhibit long-range correlations in the fluctuations of the inter-spike-interval (ISI) or spike counts within various temporal windows (Teich, 1989; Gebber et al., 2006).

Long-range correlations have been observed in spontaneous neuronal activities in various brain regions, such as the hippocampus, basal ganglia, and visual cortex (Bhattacharya et al., 2005; Darbin et al., 2006; Munn et al., 2020). Long-range correlations indicate the neural network’s “memory” of its past, suggesting that two spikes separated far away in a firing sequence are not entirely independent (Das et al., 2003; Bhattacharya et al., 2005). The presence of long-range correlations in neuronal activity indicates a “healthy” neural network, and is essential for efficient information processing in the brain (Beggs and Plenz, 2004; Kello et al., 2010; Hohlefeld et al., 2012). Studies have shown that fractal patterns with long-range correlations in neuronal firing reflect a balance between stability and excitability, representing an optimal state for neuronal information processing (Darbin et al., 2006; Hohlefeld et al., 2012). Compared to periodic or random firing, fractal firing with long-range correlations can encode more information, even when the number of action potentials remains the same over a specified time period. Previous studies have demonstrated decreased long-range correlations in the neuronal activity of patients with brain diseases such as epilepsy, Parkinson’s disease, major depression, Alzheimer’s disease and schizophrenia (Monto et al., 2007; Montez et al., 2009; Hohlefeld et al., 2012; Nikulin et al., 2012). This decrease suggests a reduction in the efficiency of information processing and is believed to be associated with pathology (Goldberger, 1996; Goldberger et al., 2002; Beggs and Plenz, 2004; Deniau et al., 2010). Therefore, the recovery of long-range correlations may serve as a biomarker reflecting the therapeutic effect in the treatment of these diseases (Hohlefeld et al., 2012; Smith et al., 2017).

Previous studies have shown that the long-range correlations can be modulated by medication or external stimulations (Linkenkaer-Hansen et al., 2004; Liang et al., 2018). For example, acupuncture stimulations applied to the bilateral hind limbs, which is effective to alleviate pain symptoms, has been observed to increase the long-range correlations in firing sequence of individual neurons in rat spinal dorsal horn (Chen et al., 2015). Sustained electrical pulses with a frequency of 70 Hz applied to the spinal cords have been found to increase the long-range correlations of electroencephalography (EEG) in patients under a minimally conscious state, indicating a restoration of cortical information integration (Liang et al., 2018; Ferdowsi et al., 2024; Wang et al., 2024). In addition, the increased long-range correlations induced by medication have been reported to be a new biomarker of the therapeutic effects in patients with infantile spasms or Parkinson’s disease (Hohlefeld et al., 2012; Smith et al., 2017). These findings suggest that external stimulation has the potential to increase long-range correlations in neuronal activity and the recovery of long-range correlations may serve as a new biomarker that reflects the therapeutic effect in the treatment of the disease.

Sinusoidal stimulation has shown promise in TES and DBS as an effective treatment for certain neurological disorders such as Parkinson’s disease, essential tremor, epilepsy, depression, and so on (Bikson et al., 2001; Antal and Paulus, 2013; Guo et al., 2016; Liu et al., 2018). However, the influence of sinusoidal stimulation on the long-range correlations of neuronal firing remains unclear. It is plausible to hypothesis that the therapeutic efficacy of sinusoidal stimulation in treating multiple diseases is also attributable to the modulation of long-range correlations in neuronal firing. Nevertheless, previous studies have demonstrated that sustained sinusoidal stimulation with a constant frequency can effectively modulate neuronal firing, resulting in precise time-locked firing patterns occurring at specific phases within the sinusoidal wave’s cycle (Wang et al., 2020). Long-range correlations in neuronal firing are indicative of nonlinearity and irregularity, suggesting a substantial level of variability in the spiking activity of neurons. From this perspective, it seems that sinusoidal stimulation may potentially increase the regularity and reduce the long-range correlations of neuronal firing. Therefore, a crucial and compelling question arises: does periodic sinusoidal stimulation increase or decrease the long-range correlations of neuronal firing?

To address this question, we conducted an analysis of the fractal properties of neuronal firing modulated by sinusoidal stimulation in hippocampal CA1 region in anesthetized rats. Specifically, sinusoidal stimulation was applied in the Schaffer collaterals, which are afferent fibers connected to the CA1 region. Unit spikes of both the pyramidal cells and interneurons in the downstream area of stimulation were recorded to examine the change of fractal of neuronal firing during sinusoidal stimulation by calculating the Fano factor and Hurst exponent of the spike sequence (Darbin et al., 2006; Munn et al., 2020). Results of the study can reveal novel nonlinear dynamics in the firing patterns of real individual neurons and provide essential insights into the mechanisms underlying the effects of sinusoidal stimulation.

## 2 Materials and methods

### 2.1 Animals and surgery

**Figure 1:**
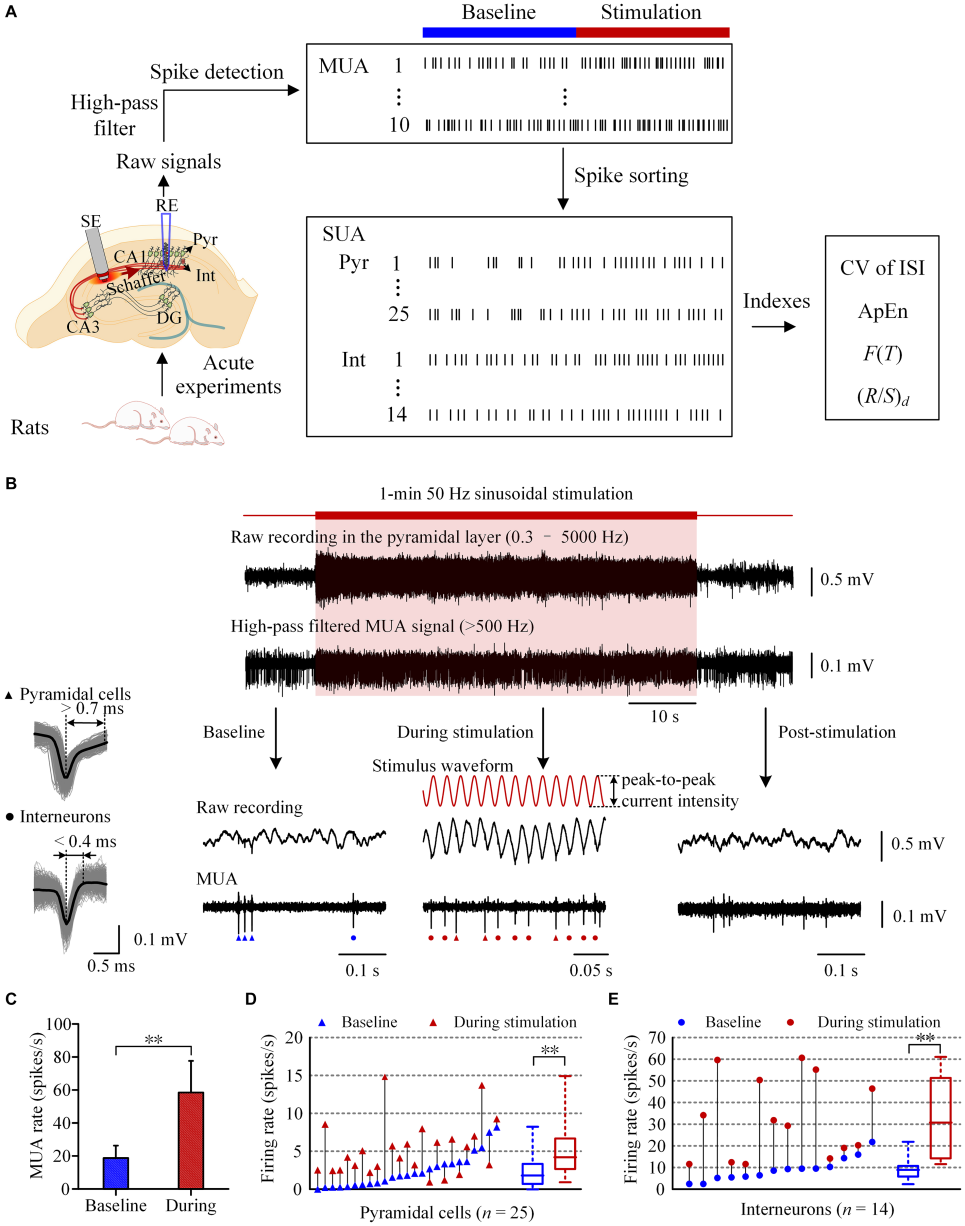
Increase of neuronal firing during the period of 50 Hz sinusoidal stimulation of Schaffer collaterals in the hippocampal CA1 region. **(A)** Workflow of the animal experiments and data acquisition, including schematic diagram of the implant positions of the recording electrode (RE) in the pyramidal layer and the stimulation electrode (SE) in the Schaffer collaterals of CA1 region. **(B)** A typical example of neuronal responses to 1-min 50 Hz sinusoidal stimulation. The shadow denotes the stimulation period. The red curves denote the sinusoidal waveforms applied at the Schaffer collaterals. Superposition traces of spike waveforms with an average waveform denoted by the black color of a typical pyramidal cell and an interneuron were shown on the *left*. Triangles and dots mark the firing of pyramidal cells and interneurons, respectively. **(C)** Comparison of the mean MUA firing rates between the baseline recordings and the recordings during stimulation. **(D,E)** Comparison of the firing rates of pyramidal cells and interneurons between the baseline recordings and the recordings during stimulation. Baseline recordings were obtained 1-min before the sinusoidal stimulation. **p < 0.01, paired t-test.

The animal experiment was approved by the Laboratory Animal Welfare and Ethics Committee of Zhejiang University (Ethics Code: ZJU20210108). The data were obtained from 10 adult male Sprague–Dawley rats (325 ± 27 g), utilizing previously established methodologies (Feng et al., 2013, 2017). The rats were anesthetized with urethane (1.25 g/kg, i.p.) and fixed in a stereotaxic frame (Stoelting Co., United States). A partial craniotomy was performed to allow the placement of electrodes. A16-channel silicon electrode probe (#Poly2, NeuroNexus Technologies, United States) was inserted into the left hippocampal CA1 region (AP −3.5 mm; ML 2.7 mm; DV ~2.5 mm) as a recording probe. A concentric bipolar stainless-steel electrode (#CBCSG75, FHC Inc., United States) was inserted into the afferent axons (i.e., the Schaffer collaterals) of the CA1 region (AP −2.2 mm; ML 2.2 mm; DV ~2.8 mm) to apply orthodromic stimulation to the upstream of the recording probe (Figure 1A). Four neighboring contacts in the recording probe located in pyramidal layer of CA1 region were utilized to collect unit spikes. Two stainless steel screws were anchored into the nasal bone, serving as the reference and ground electrodes, respectively. The accuracy of electrodes placement was confirmed based on the sequential appearance of unit spike signals in the recording array and the potential waveform induced by pulse stimulation, as previously described (Kloosterman et al., 2001).

### 2.2 Stimulation and recording

Sinusoidal waveforms were generated using a signal generator (DG1032Z, REGOL Technologies, China) and converted into symmetrical sinusoidal stimulus currents via an analog stimulus isolator (Model 2200, A-M Systems Inc., United States). The currents were delivered to the rat brain through a stimulation electrode. The frequency of sinusoidal stimulation was 50 Hz. The peak-to-peak current intensity of the sinusoidal waveforms was adjusted to 35–65 μA (53.0 ± 8.9 μA, 10 rats) to modulate the unit firing of neurons in downstream without evoking large amplitude population spikes resulted from synchronized neuronal firing. The duration of the sinusoidal stimulation was 1 min.

The extracellular electrical signals recorded by the electrode were amplified 100-fold using a microelectrode amplifier (Model 3600, A-M Systems Inc., United States) and passed through a band-pass filtering with a range of 0.3–5,000 Hz. The signals were then sampled at 20 kHz using a PowerLab data acquisition system (Model PL3516, AD Instruments Inc., Australia) and stored for off-line analysis.

### 2.3 Analysis of unit spikes

Figure 1A illustrates the process of acute experiments and data acquisition. A LabChart8 (AD Instruments Inc., Australia) built-in digital high-pass filter (>500 Hz) was used to remove the 50 Hz sinusoidal stimulation artifacts and local field potential (LFP) in raw recording signals, and to generate multiple unit activity (MUA) signals. The lower cut-off frequency was set at 500 Hz to gain better spike detection and sorting effects (Feng et al., 2012). MUA signals from four neighboring contacts of the recording electrode array in the pyramidal layer of the CA1 region were utilized to extract single unit activity (SUA) of pyramidal cells and interneurons. Spike detection and sorting were conducted in accordance with established methods (Feng et al., 2017). Finally, the spike sequence of each individual neuron was obtained, and the ISI sequence was then acquired accordingly.

To examine the effect of axonal sinusoidal stimulation on downstream neurons, the mean firing rates of MUA and SUA, as well as the coefficient of variation (CV) of ISI of spike sequences were calculated during 1-min sustained sinusoidal stimulation and in 1-min baseline recording before stimulation as a control. The Approximate Entropy (ApEn), a metric previously used to evaluate EEG and neuronal firing complexity, was calculated to evaluate the statistical irregularity of neuronal firing (Sleigh and Donovan, 1999; Natarajan et al., 2004). The calculation of ApEn followed established methods, with an embedding dimension (*m*) of 2 and a vector comparison length (*r*) of 0.15 (Sleigh and Donovan, 1999; Natarajan et al., 2004). The use of a small *m* and moderate *r* ensures the reliability of ApEn and facilitates more accurate comparisons between different data groups. Low ApEn values indicate low irregularity, whereas high ApEn values signify high irregularity. The Fano factor and Hurst exponent were calculated to evaluate the changes of long-range correlations (one of fractal properties) in spike sequences of individual neurons (Darbin et al., 2006; Munn et al., 2020).

The Fano factor, abbreviated as *F*(*T*), is defined as the ratio of the variance to the mean of spike counts within a time window of duration *T* (in second) (Equation 1):


(1) $$F\left(T\right)=\frac{\mathrm{var}\left[N_{i}\left(T\right)\right]}{\mathrm{mean}\left[N_{i}\left(T\right)\right]}$$

For the recordings of 1-min sinusoidal stimulation and 1-min baseline, *T* is increased from a minimum of 0.1 s to a maximum of 10 s. *N_i_*(*T*) represents the spike counts in the *i*^th^ window of size *T*. The Fano factor curve *F*(*T*) is plotted using double-logarithmic scales.

In spike sequences with long-range correlations, the *F*(*T*) increases as a power-law function of the window size and may exceed 1.0. This increase reflects the greater variance in spike counts with larger window sizes (Teich, 1989; Lowen et al., 2001). The increased variance results from the presence of infrequent clusters of long and short ISIs, which become more apt to be found with increased data collection. Such clustering is a characteristic feature of fractal processes (Teich, 1989). On a double-logarithmic scale, the power-law relationship between *F*(*T*) and window size (*T*) appears as a straight line. Subsequently, the slope α of the Fano factor time curve was calculated to estimate of the fractal scaling exponent (Munn et al., 2020). If the neuronal firing is a Poisson process in which the ISIs are uncorrelated, *F*(*T*) would be approximately 1.0 for all *T* because the variance of a Poisson process is equal to the mean. If the neuronal firing is a periodic process, its *F*(*T*) would approach 0 because the variance decreases as the window size increases.

The second index of long-range correlations, Hurst exponents (*H*), is defined on the ISIs of spike sequences by the following rescaled range method (Equations 2–4) (Hurst, 1951; Darbin et al., 2006). Firstly, the ISI sequences with a total ISI number of *N* are divided into *M* adjacent segments, denoted as *I_m_*, each with an ISI number of of *d*. Here *M* × *d* = *N* and *m* = 1,…, *M.* Next, for each segments *I_m_*, the mean (*E_m_*) and standard deviation (*S_m_*) of ISIs are calculated. The accumulated deviation from the mean is then computed for each segment *I_m_*, which is defined as the running sum of the differences between individual ISIs and the mean value:


(2) $$X_{k,m}=\overset{k}{\underset{i=1}{\sum }}\left(\mathrm{IS}I_{i,m}-E_{m}\right)\;k=1,2,\ldots ,d\text{.}$$

The rescale range *R_m_*, is defined as the difference between the maximum and minimum values of the accumulated deviation:


(3) $$R_{m}=\max\left(X_{k,m}\right)-\min\left(X_{k,m}\right),k=1,2,\ldots ,d\text{.}$$

where *X*_*k*,*m*_ is actually the vector [*X*_*1*,*m*_, *X*_*2*,*m*_,…, *X_d,m_*].

Thus, the mean value of the rescaled range for all segments of length *d* were calculated:


(4) $${\left(R/S\right)}_{d}=\frac{1}{M}\overset{M}{\underset{m=1}{\sum }}R_{m}/S_{m}$$

The length *d* is increased from a minimum of 4 to a maximum of *N*/3. Finally, the Hurst exponent (*H*) is estimated from the slope of double-logarithmic plots of (*R*/*S*)*_d_* against *d*. The value of *H*, ranging from 0 to 1.0, determines whether the spike sequences exhibit fractal properties. If *H* is not equal to 0.5, neuronal firing is long-range correlated (Jackson, 2004; Nurujjaman et al., 2009). When 0 < *H* < 0.5, the spike sequence exhibits negative long-range correlations: a decrease of ISI is typically followed by an increase, and vice versa. Conversely, a range of 0.5 < *H* < 1 indicates positive long-range correlations: a decrease of ISI leads to further decreases of ISI, while an increase leads to more increases. When *H* = 0.5, successive changes in ISIs become independent, indicating no long-range correlations. Therefore, *H* can indicate the directions of long-range correlations, while the α value of *F*(*T*) cannot.

The data are presented as the mean ± standard deviation (SD), with “*n*” representing the number of neurons. Based on the results of Shapiro–Wilk tests that the data were normally distributed, the statistical significance of the differences between the groups of that during 1-min sustained sinusoidal stimulation and at the baseline recording was tested by paired *t*-test.

## 3 Results

### 3.1 Increase of neuronal firing by sustained sinusoidal stimulation of afferent axons in the CA1 region

During sustained 50 Hz sinusoidal stimulation applied at the Schaffer collaterals, the signals collected in the CA1 pyramidal layer comprised MUA, LFP, and sinusoidal stimulation artifacts (Figures 1A,B). By applying a digital high-pass filter (>500 Hz) to the recorded signals, the MUA signals were extracted (Figure 1B). The extracted MUA signals effectively demonstrated an increase in neuronal firing in the downstream region during the 1-min 50 Hz sinusoidal stimulation period. The mean firing rate of MUA during stimulation period (58.4 ± 19.2 spikes/s) was significantly higher than that at baseline recording 1-min before stimulation (18.7 ± 7.6 spikes/s; *p < 0.01, paired t-test.* *n* = 10 rats, Figure 1C). After the termination of the stimulation, the MUA signals promptly declined and then returned to baseline levels. The MUA signals recorded in CA1 region consisted of SUA of pyramidal cells and interneurons. We next investigated the effects of sinusoidal electrical stimulation on the firing of different kinds of neurons.

In 10 rat experiments, we obtained SUA from a total of 39 neurons in the CA1 pyramidal layer, including 25 pyramidal cells and 14 interneurons. During 1-min 50 Hz sinusoidal stimulation, the firing rate of pyramidal cells significantly increased from 2.4 ± 2.2 spikes/s at baseline to 5.2 ± 3.5 spikes/s during stimulation period (*p < 0.01, paired t-test.* *n* = 25, Figure 1D). Similarly, the firing rate of interneurons also increased significantly from 9.1 ± 5.4 spikes/s at baseline to 32.4 ± 18.8 spikes/s during the stimulation period (*p < 0.01, paired t-test.* *n* = 14, Figure 1E). The firing rates of both pyramidal cells and interneurons were significantly less than the stimulation frequency of 50 Hz during stimulation period. The coupling ratio (firing rate / stimulation frequency) was approximately 10% for pyramidal cells and 65% for interneurons. In addition, the firing rate of interneurons was significantly higher than that of pyramidal cells, both at baseline and during stimulation (*p* < 0.01, *t*-test).

These results indicate that 50 Hz sinusoidal stimulation at the Schaffer collaterals can activate both pyramidal cells and interneurons in the downstream CA1 region. However, the neurons failed to follow every cycle of the sinusoidal stimulation. Then, did their firing patterns change as well? We next investigated the effect of sinusoidal stimulation on the firing pattern of downstream pyramidal cells and interneurons by analyzing the ISI of these neurons.

### 3.2 Changes of firing patterns of CA1 neurons during sustained sinusoidal stimulation

**Figure 2:**
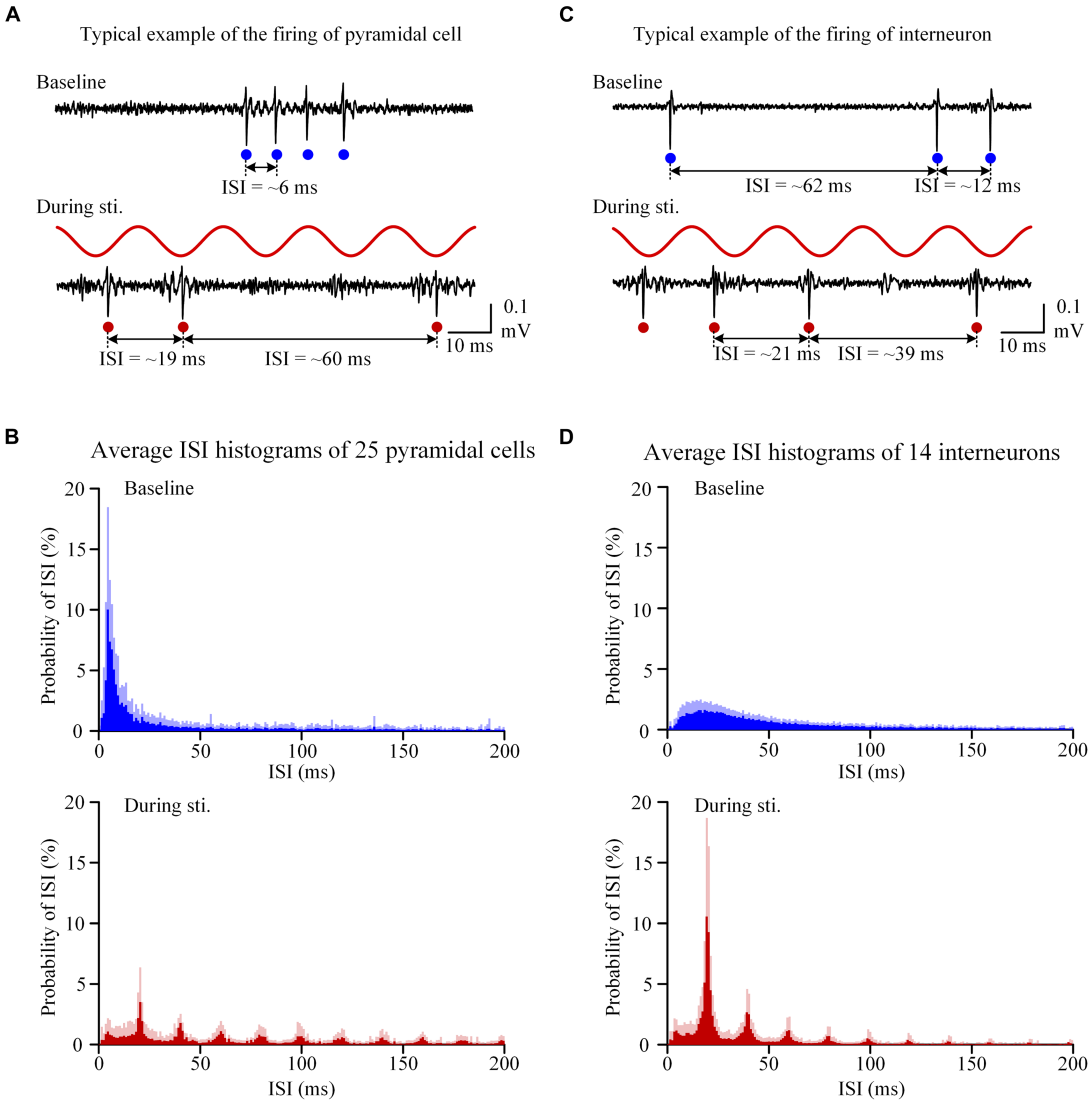
Sinusoidal stimulation induced changes of firing patterns of both pyramidal cells and interneurons. **(A)** A typical example of unit spike recording of pyramidal cells before and during stimulation. **(B)** The average inter-spike-interval (ISI) curve of 25 pyramidal cells. **(C,D)** Corresponding plots as panels **(A,B)** for interneurons with a same order in panels **(A,B)** but neuronal types changed from pyramidal cells to interneurons. On the recording signals, red and blue dots denote unit spikes at baseline and during stimulation, respectively.

At baseline recording before stimulation, the unit spikes of pyramidal cells exhibited burst firing patterns, characterized by consecutive spikes with a short ISI less than 8 ms (Figure 2A). The bursty character resulted in an obvious peak (~ 5 ms) in the ISI histogram and approximately 35% of ISIs was in the range of 0–8 ms (Figures 2A,B). During the 1-min stimulation period, unit spikes of the pyramidal cells were typically generated separately near the negative peak of the sinusoidal stimulation waveform. As a result, most of the ISIs were close to integer multiples of the 20 ms period of 50 Hz sinusoidal stimulation (Figure 2B). The probability of ISIs in the range of 0–8 ms during stimulation was significantly lower than baseline recording (Table 1). However, distinct peaks appeared at *n*-fold multiples of the 20 ms periods of the 50 Hz sinusoidal stimulation (Figure 2B). For instance, the probability of ISIs falling within 19–21 ms (i.e., approximately one period of the 50 Hz stimulation) was significantly higher during stimulation compared to the corresponding baseline value (Table 1). The distribution of unit spikes of interneurons was more scattered than that of pyramidal cells at baseline recording (Figure 2C). Consequently, only 5.3 ± 3.8% of the ISIs were within the range of 0–8 ms, similar to the corresponding value of 6.1 ± 5.8% observed during stimulation (Table 1). Additionally, similar to pyramidal cells, ISI peaks were observed at *n*-folds of the 20 ms periods of 50 Hz sinusoidal stimulation. Notably, the probability of ISIs within the first peak (19–21 ms) of the ISI histogram was significantly higher than the corresponding baseline value (Table 1).

**Table 1:** Comparison of distribution, CV, and ApEn of the ISI of spike sequences.

Cell type	Number of cells	Group	ISI in the range of 0–8 ms	ISI in the range of 19–21 ms	CV of ISI	ApEn
Pyramidal cell	25	Baseline	34.3 ± 22.5%	2.5 ± 2.4%	0.64 ± 0.22	0.87 ± 0.28
		During sti.	8.7 ± 9.5% ^a^	7.3 ± 7.6% ^a^	0.63 ± 0.36	1.05 ± 0.32^a^
Interneuron	14	Baseline	5.3 ± 3.8%	2.5 ± 2.4%	0.73 ± 0.17	1.16 ± 0.18
		During sti.	6.1 ± 5.8%	23.9 ± 15.8% ^a^	1.33 ± 0.59 ^a^	1.33 ± 0.26^a^

a Baseline vs. During sti.

*p < 0.01, paired t-test.*

To better understand the changes in firing pattern of pyramidal cells and interneurons during stimulation, we next calculated the CV and ApEn of the ISIs. The CV of ISI of interneurons during stimulation was significantly larger compared to the corresponding baseline value (Table 1). However, despite the transformation of the ISI distribution from a single-peak to a multi-peaks distribution during stimulation, the CV of ISI of pyramidal cells showed no significant difference between the baseline and stimulation period (Table 1). The mean ApEn of pyramidal cells increased from 0.87 ± 0.28 at baseline to 1.05 ± 0.32 during stimulation and the mean ApEn of interneurons also increased from 1.16 ± 0.18 at baseline to 1.33 ± 0.26 during stimulation (Table 1). Higher ApEn values indicate higher irregularity. The irregularity could be caused by long-range correlations among spikes or by random disturbance from circumstances without history-effect or memory. Next, we calculated the Fano factor *F*(*T*) and the Hurst exponent (*H*) of the spike sequences to compare the firing patterns and long-range correlations of neuronal firing at baseline and during stimulation.

### 3.3 Changes of long-range correlations in the neuronal firing during sustained sinusoidal stimulation

**Figure 3:**
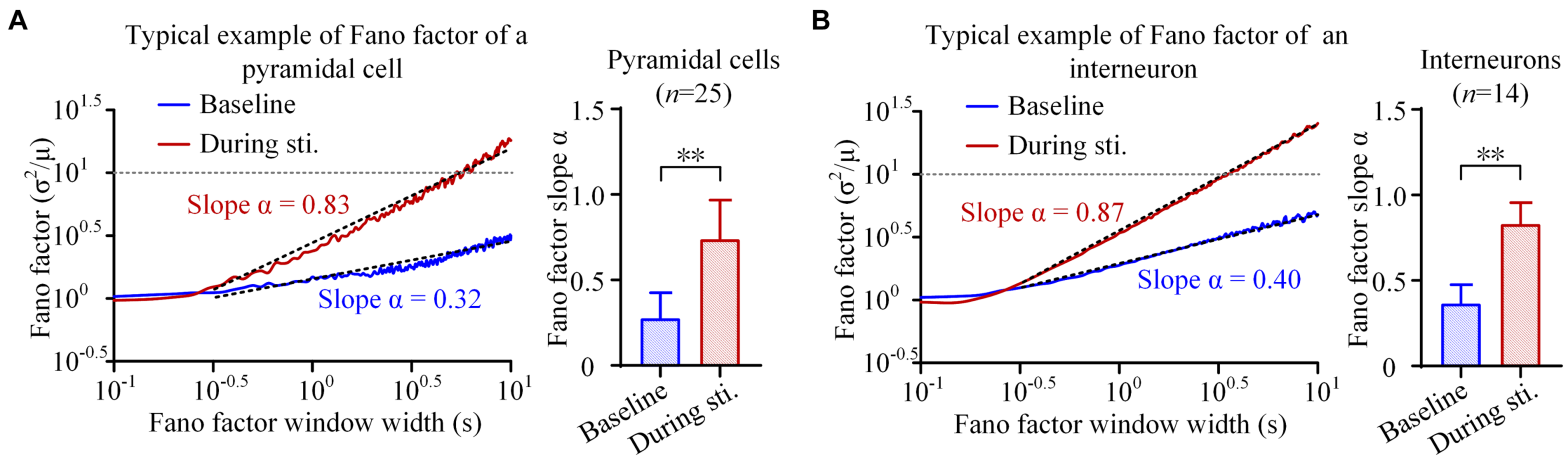
Fano factor analyses of the firing of pyramidal cells and interneurons. **(A)** *Left*: The average Fano factor plots of the total 25 pyramidal cells. Fano factors were expressed as function of the time lags (in seconds, double logarithmic scales). *Right*: The comparison of scaling exponent of Fano factor curve between the baseline recordings of pre-stimulation and the recordings during stimulation. **(B)** The Fano factor plots and comparison of scaling exponent of interneurons. **p < 0.01, paired t-test.

During 1-min sinusoidal stimulation, the Fano factor of pyramidal cells increased with an increase in window size, reaching a maximum value exceeding 10 (Figure 3A). Importantly, a power law relationship was observed starting at a window size near 10^–0.5^ s (~0.32 s), resulting in a straight line with a positive slope of approximately 0.8 in the double-logarithmic plot (Figure 3A). At baseline recording, the Fano factor of pyramidal cells also increased with an increase in window size, and showed a power law relationship starting at a window size near 10^–0.5^ s (~0.32 s). However, both the rate of increase and the maximum value of Fano factor at baseline recording were lower compared to those observed during stimulation (Figure 3A). The slope *α* of the Fano factor during stimulation was 0.74 ± 0.23, which was significantly higher than the corresponding value of 0.28 ± 0.15 at baseline (*p < 0.01, paired t-test.* *n* = 25, Figure 3A). Similarly, within the window size in the range of 10^–0.5^ s (~0.32 s) to 10^1^ s (~10 s), Fano-factor curves of interneurons also exhibited a power law relationship with a straight line in double-logarithmic plots, both at baseline and during stimulation (Figure 3B). The slope α of interneurons during stimulation was 0.83 ± 0.12, approaching its maximum theoretical value of 1.0 and significantly higher than the baseline value of 0.36 ± 0.11 (*p < 0.01, paired t-test.* *n* = 14, Figure 3B). The power law relationship observed in the Fano factor curves suggests the presence of long-range correlations in the firing of both pyramidal cells and interneurons. The larger slope α during stimulation indicates the long-range correlations were stronger during sinusoidal stimulation than at baseline.

**Figure 4:**
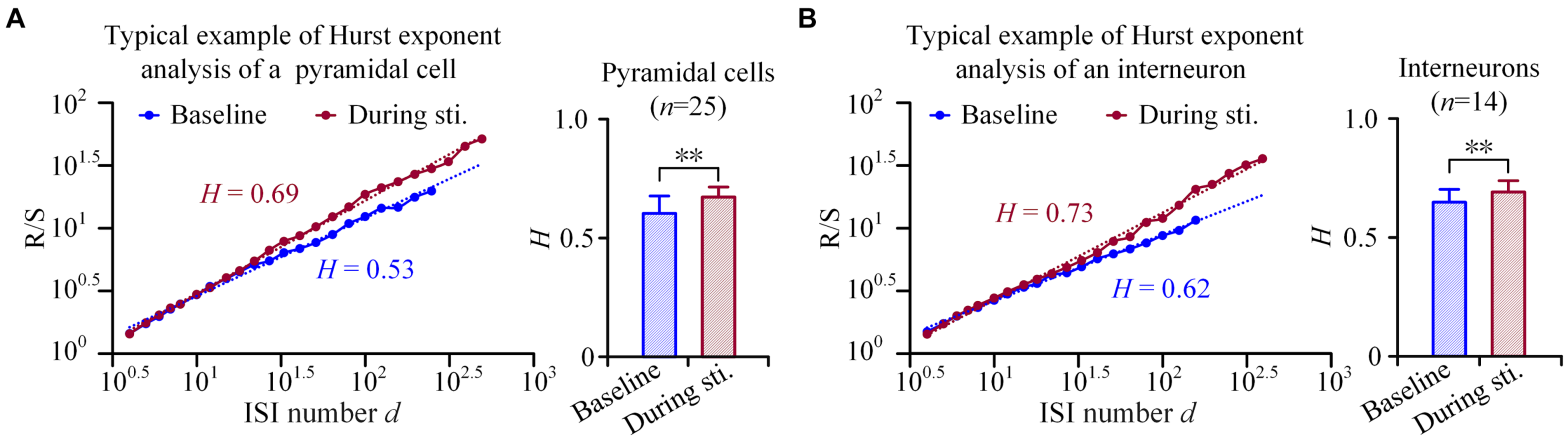
Hurst exponents analyses of the firing of pyramidal cells and interneurons. **(A)** *Left*: Examples of rescale range (Hurst exponents) analyses of a pyramidal cell. The image shows a linear relationship between (*R*/*S*)*_d_* and the ISI number *d*, when plotted in double-logarithmic representation. *Right*: Comparison of the Hurst exponent of pyramidal cells between the baseline recordings of pre-stimulation and the recordings during stimulation. **(B)** Corresponding plots as panel **(A)** for interneurons. **p < 0.01, paired t-test.

These findings were further confirmed by the rescaled range analysis. In the double-logarithmic plots of (*R*/*S*)*_d_* against segments length with ISI number *d*, both pyramidal cells and interneurons displayed an increase in R/S value with an increase in the ISI number *d*, both during stimulation and at baseline (Figures 4A,B). Although both types of neurons exhibited a power law relationship characterized by a straight line in the double-logarithmic plots of (*R*/*S*)*_d_* against *d*, the mean *H* value [i.e., the slope of (*R*/*S*)*_d_*] during stimulation was larger compared to baseline. Specifically, during the 1-min 50 Hz sinusoidal stimulation, the *H* value of pyramidal cells and interneurons significantly increased from 0.60 ± 0.07 and 0.64 ± 0.05 at baseline to 0.67 ± 0.04 and 0.69 ± 0.04 during stimulation, respectively (*p < 0.01, paired t-test.* *n* = 25 for pyramidal cells and *n* = 14 for interneurons, Figures 4A,B). An *H* value greater than 0.5 indicates positive long-range correlations among spikes, and an increase in *H* value during sinusoidal stimulation further enhances the positivity in the long-range correlations.

These results indicated that the periodic sinusoidal stimulation without long-range correlations can actually increase the long-range correlations in neuronal firing of both pyramidal cells and interneurons.

## 4 Discussion

The major finding of this study is that the sustained periodic axonal sinusoidal stimulation without long-range correlations can recruit the firing and increase rather than decrease the long-range correlations in the firing of both pyramidal cells and interneurons in the post-synaptic area downstream the stimulation site in rat hippocampus. To our knowledge, this is the first study demonstrating the change of fractal properties of neuronal firing induced by sinusoidal stimulation. The findings and their implications are discussed in the following text.

When a single sinusoidal wave was applied to the neural tissue, its negative phase (i.e., negative half-period) can induce depolarization of axonal membranes, leading to the generation of an action potential. The action potential then propagates along the axons to the axon terminals, and subsequently, transmits across the synapses to activate the pyramidal cells and interneurons in the post-synaptic CA1 region. However, when the stimulus was repeated at a high frequency, the downstream neurons usually cannot follow each stimulus to fire action potential. Firstly, it is possible that the axons directly evoked by sinusoidal waveform generated action potentials at a rate less than the 50 Hz stimulation frequency. Secondly, previous studies have shown that 50 Hz sinusoidal stimulation at axons can induce axonal conduction block, which may be caused by the extracellular K^+^ concentration elevation, the intracellular Na^+^ accumulation as well as the slow recovery of Na^+^ channels (Jensen and Durand, 2007; Bellinger et al., 2008; Zang and Marder, 2021; Zang et al., 2023). Moreover, the possible effects of synaptic failure and neurotransmitter depletion in the process of synaptic transmission may also attenuate the excitatory effect of stimulation on downstream neurons (Kim et al., 2012; Rosenbaum et al., 2014). These effects prevent both pyramidal cells and interneurons in the downstream CA1 region from following each stimulation cycle to generate action potentials. Consequently, the firing rates of both pyramidal cells and interneurons were less than stimulation frequency of 50 Hz, and peaks appeared at *n*-folds of the 20 ms periods of 50 Hz sinusoidal stimulation in the ISI histogram (Figures 1, 2). The ISIs transferring from random values to 20 ms or *n*-folds of the 20 ms during stimulation also indicated that 50 Hz sinusoidal stimulation can modulate the spontaneous firing of pyramidal cells and interneurons to a stimulation-related pattern.

An intriguing finding of this study is that the sustained periodic sinusoidal stimulation inputs, without long-range correlations, induced fractal rather than random or regular firing patterns in the downstream region. This phenomenon can be attributed to the intrinsic properties of neurons and the mechanism of stimulation-induced axonal block and synaptic failure. Previous studies have demonstrated that the biophysical origin of long-range correlations may arise from the intrinsic properties of ion channels and synapses in neurons (Teich et al., 1997; Darbin et al., 2006). It has been reported that the opening and closing of ion-channels, such as Na^+^ and K^+^ channels, in neuronal membranes showed long-range correlations (Liebovitch and Czegledy, 1992; Lowen et al., 1999). During sustained axonal sinusoidal stimulation, the intense activations from sinusoidal stimulation can elevate the concentration of K^+^ in the peri-axonal spaces, thereby raising the membrane potential to a depolarization level that leads to intermittent axonal block (Bellinger et al., 2008; Guo et al., 2018). In addition, the intracellular accumulation of Na^+^ and slow recovery of Na^+^ channels may also contribute to action potential conduction failure (Zang and Marder, 2021; Zang et al., 2023). However, even at this elevated potential, the dynamics of Na^+^ and K^+^ channels remain followed their intrinsic nonlinear properties, initiating action potentials at an even more nonlinear level (Hodgkin and Huxley, 1952). Consequently, periodic sinusoidal stimulation can activate axonal fibers in a fractal pattern and increase the long-range correlations in the induced firing patterns. Fractal patterns have also been observed in the secretion of neurotransmitters from the pre-synaptic membrane (Lamanna et al., 2015). These fractal properties may lead to a fractal pattern in the recovery from stimulation-induced synaptic failure and neurotransmitter depletion. Fractal excitation of axons induced by periodic sinusoidal stimulation, requires synaptic transmission for its propagation to downstream neurons. The fractal properties of synapses may further contribute to the generation of fractal impulses in the downstream neurons. Additionally, neuronal network interactions may also contribute to fractal neuronal firing (Teich, 1989). For example, the fractal neuronal firing in the sensory areas of brain cortex, which propagates to the basal ganglia, can induce fractal neuronal firing in substantia nigra cells (Teich et al., 1997; Rodriguez et al., 2003; Darbin et al., 2006). In our experimental investigations of the hippocampal CA1 region, pyramidal cells and interneurons form densely interconnected local circuits involving feedforward and feedback inhibitions (Andersen et al., 2007; Ahmed and Mehta, 2009). The interactions among pyramidal cells and interneurons may contribute to the increased fractal properties of neuronal firing during stimulation.

Consequently, the neuronal firing induced by sustained periodic sinusoidal stimulation exhibited a fractal pattern rather than a periodic firing pattern with constant ISI. Downstream neurons may respond to two or more adjacent sinusoidal cycles, resulting in the formation of spike clusters with an ISI of 20 ms. It is also possible that two or more consecutive cycles of sinusoidal stimulation failed to induce action potentials in downstream neurons, leading to the formation of larger spike clusters with ISIs that were twice (40 ms) or *n*-folds of the 20 ms period of 50 Hz sinusoidal stimulation. These spike clusters, varying in ISIs from 20 ms to *n* × 20 ms, exhibited a nested organization, where smaller time-scale subclusters being parts of larger clusters that appear on larger time scales. This nested organizational structure may explain the enhanced long-range correlations of neuronal firing during stimulation.

Previous studies have demonstrated that epilepsy, Parkinson’s disease, Alzheimer’s disease, schizophrenia, major depressive disorder, post-traumatic stress disorder, and age-related cognitive disorders are associated with impaired long-range correlations in neuronal activity (Monto et al., 2007; Montez et al., 2009; Hohlefeld et al., 2012; Nikulin et al., 2012; Mishra and Gazzaley, 2014; Dimitriadis and Linden, 2016). A decrease in long-range correlations indicates disrupted temporal patterning of neuronal activity and is believed to be associated with pathological processes (Goldberger et al., 2002; Berendse and Stam, 2007; Hohlefeld et al., 2012). The increase of long-range correlations in LFP and EEG recordings from patient under DBS, SCS and closed-loop neurofeedback stimulation has been observed to correlate with therapeutic efficacy (Hohlefeld et al., 2012; Hohlefeld et al., 2013; Zhigalov et al., 2016; Smith et al., 2017; Liang et al., 2018). For instance, an effective therapy of thalamic DBS has been found to increase the long-range correlations in the high beta-band (21–30 Hz) of EEG recording in patients suffering from essential tremor (Hohlefeld et al., 2013). Therefore, increasing long-range correlations in neuronal firing induced by sinusoidal stimulation may be significant for suppressing pathological firing patterns and achieving therapeutic effects.

Furthermore, long-range correlations in the neuronal activity have been reported to reflect a balance between excitation and inhibition, which is crucial for optimal information processing in the brain (Kello et al., 2010; Hohlefeld et al., 2013; Liang et al., 2018). Therefore, the stimulation induced increase of long-range correlations may represent an optimal state for information processing. The fractal properties of neuronal firing mean the existence of a “memory” or historical influence on the timing of neuronal firing. Specifically, the timing of a particular spike is partly determined by the timing of previous spikes from the same neuron (Bhattacharya et al., 2005). The historical effect results in the neuronal firing fluctuating across multiple time scales. The fluctuations offer potential benefits, including facilitating flexible and efficient information coding and enhancing error tolerance during encoding (Voss, 1992; West, 2013). Moreover, the fluctuations allow the neuronal system with the capacity to adapt flexibly to new and demanding external perturbations (Bhattacharya et al., 2005). Therefore, the increase of long-range correlations induced by sinusoidal stimulation reflects its potential benefit in neuronal information processing.

According to previous research, stimulation frequency is a key factor affecting the firing activities of neurons (Bello et al., 2020). Stimulation at lower frequencies can induce reliable propagation of action potentials, while stimulation at kilohertz frequency can totally block action potential conduction on axons (Kilgore and Bhadra, 2014). Therefore, further research is necessary to compare the modulatory effects of different stimulation frequencies on the long-range correlations of neuronal firing. Moreover, additional studies are needed to replicate the findings in different brain regions (e.g., basal ganglia) in order to establish the universality of sinusoidal stimulation on neurons. Finally, further investigations are essential to validate the therapeutic efficacy of sinusoidal stimulation in pathological animal models.

## 5 Conclusion

The current study firstly demonstrates that sinusoidal stimulations of afferent axonal fibers with a fixed frequency can modulate neuronal firing and enhance long-range correlations of neuronal firing in rat hippocampal CA1 region. The finding suggests that sinusoidal stimulation may be an alternative waveform in brain stimulation. It also provides a new potential mechanism for the therapeutic effects of sinusoidal stimulation to improve instead of to destroy the information processing of brain.

## Data Availability

The original contributions presented in the study are included in the article/supplementary material, further inquiries can be directed to the corresponding author.
